# Quantum Adiabatic Pumping in Rashba- Dresselhaus-Aharonov-Bohm Interferometer

**DOI:** 10.3390/e21090828

**Published:** 2019-08-24

**Authors:** Yasuhiro Tokura

**Affiliations:** 1 Faculty of Pure and Applied Sciences, University of Tsukuba, 1-1-1, Tennodai, Tsukuba, Ibaraki 305-8571, Japan; tokura.yasuhiro.ft@u.tsukuba.ac.jp; 2 Tsukuba Research Center for Energy Materials Science (TREMS), University of Tsukuba, 1-1-1, Tennodai Tsukuba, Ibaraki 305-8571, Japan

**Keywords:** spin pump, spin-orbit interaction, quantum adiabatic pump, interferometer, geometric phase

## Abstract

We investigate the quantum adiabatic pumping effect in an interferometer attached to two one-dimensional leads. The interferometer is subjected to an Aharonov-Bohm flux and Rashba-Dresselhaus spin-orbit interaction. Using Brouwer’s formula and rigorous scattering eigenstates, we obtained the general closed formula for the pumping Berry curvatures depending on spin for general interferometers when the external control parameters only modulate the scattering eigenstates and corresponding eigenvalues. In this situation, pumping effect is absent in the combination of the control parameters of Aharonov-Bohm flux and spin-orbit interaction strength. We have shown that finite pumping is possible by modulating both Rashba and Dresselhaus interaction strengths and explicitly demonstrated the spin-pumping effect in a diamond-shaped interferometer made of four sites.

## 1. Introduction

Coherent transport in mesoscopic systems is of fundamental interest since it allows realization of various phenomena observed in quantum optics in a solid-state system. Furthermore, the electron spin degree of freedom adds an intriguing knob for the manipulation and observation of the transport phenomena. Spin-orbit interaction (SOI) effect [1] is one of the key ingredients in narrow-gap semiconductor devices, whose strength can be controlled by external gates [2], in principle, without changing the electron density. Introducing the effect of SOI to the electron interferometer structure is quite attractive since it enables perfect spin filtering effect [3,4,5]. Moreover, transient behavior in such an interferometer has been investigated [6].

In addition to passive functional devices such as filters, the active functions, for example, spin-pumping or spin manipulation effect by dynamically modulating the gate voltages [7,8,9], magnetic field [10,11,12,13], or magnetization of the ferromagnets [14,15,16,17], has been investigated. In particular, quantum adiabatic pumping (QAP) phenomena [18,19], which stems from geometrical properties of the dynamics, is an active field of research [20,21,22,23,24,25]. In the non-interacting limit, QAP is related to the scattering matrix of the coherent transport. We have investigated the QAP effect by adiabatically modulating the Aharonov-Bohm (AB) phase [26] of the interferometer as well as the local potential in the interferometer. However, it seems no studies have been made of the adiabatic spin-pumping with purely geometric means such as Aharonov-Casher phase or AB phase. The fundamental question here is whether QAP is possible by only modulating the electron geometric phase.

In this work, we studied spin-QAP in Rashba-Dresselhaus-Aharonov-Bohm interferometer introduced in [3] using Brouwer’s formula [19] and derived an explicit formula of the Berry curvature for each spin component. Using the obtained result, we clarified the condition of finite spin-pumping. In Section 2, we introduce a simple two-terminal setup and the expressions of the scattering amplitudes. Section 3 explains the details of the eigenstates of the scattering problem. Then, with these states, the formula of the QAP is derived in Section 4. It is shown that the modulation of the AB phase cannot induce QAP. Section 5 explains the properties of the diamond-shape interferometer, and is applied to study QAP assuming Rashba SOI and Dresselhaus SOI strengths as control parameters in Section 6. Finally, discussions follow in Section 7 and Appendices are included for the detailed derivations of the formula used in the main text.

**Figure 1:**
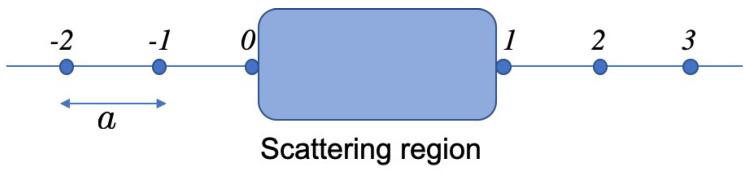
Schematics of the model of a scattering (shaded) region connected with two semi-infinite one-dimensional leads.

We consider a standard setup of scattering problem of spin 1/2 electrons as shown in Figure 1. A coherent scattering region (interferometer) is attached at the site u=0 with the one-dimensional left lead made of sites u=−1,−2,… and is attached at the site u=1 with the one-dimensional right lead made of sites u=2,3,…. The assumption of one-dimensional leads is not essential as far as the interferometer is coupled to the leads via single mode scattering channels. However, the one-dimensional tight-binding formalism benefits from its simplicity. Although the analysis is standard, the obtained rigorous scattering amplitudes and corresponding scattering eigenstates are essential to clarify the condition and to quantify the quantum adiabatic spin-pumping, as will be shown in the later sections. We introduce the spinor ket vector at site *u*,

(1) $$\begin{array}{ccc} |\psi (u)\rangle & \equiv & \left(\begin{array}{c} c_{u↑}\\ c_{u↓} \end{array}\right), \end{array}$$

where the two amplitudes $c_{u\sigma }$ for spin σ=↑,↓ satisfy normalization condition ${\left|c_{u↑}\right|}^{2}+{\left|c_{u↓}\right|}^{2}=1$. The total Hamiltonian in the tight-binding approximation is given in general

(2) $$\begin{array}{ccc} {\hat{H}}_{\mathrm{TB}} & \equiv & \sum_{u}\epsilon_{u}|\psi (u)\rangle \langle \psi (u)|+\sum_{uv}{\hat{W}}_{uv}|\psi (v)\rangle \langle \psi (u)|, \end{array}$$

where the site index *u* and *v* run the entire system. The real parameter $\epsilon_{u}$ is spin-degenerate site energy and ${\hat{W}}_{uv}$ is a 2×2 hopping matrix satisfying ${\hat{W}}_{uv}^{†}={\hat{W}}_{vu}$. We assume that the hopping matrix ${\hat{W}}_{uv}$ is only non-diagonal in the scattering region between u=0 and u=1. We neglect the electron-electron interaction.

## 2. Model System

In the leads u≤−1, u≥2, we set $\epsilon_{u}=0$ and ${\hat{W}}_{uv}=-jI$, where I is the two-dimensional unit matrix and the real hopping parameter *j* is only nonzero for nearest-neighbor pair of u,v. With a standard treatment on the tight-binding Hamiltonian, we obtain the eigen-energy $\epsilon_{k}\equiv -2j\cos\left(ka\right)$ and its corresponding eigen-function, $|\psi (u)\rangle \propto e^{ikau}|\chi \rangle$, where *k* is a real wave-number parameter, a(>0) is the lattice constant and |χ〉 is a certain state vector.

The system of interferometer is represented between u=0 and u=1 sites and we choose $\epsilon_{0}=y_{0}$ and $\epsilon_{1}=y_{1}$ and ${\hat{W}}_{01}\equiv \hat{W}$ and ${\hat{W}}_{10}={\hat{W}}^{†}$. The microscopic derivations of $y_{0},y_{1}$ and $\hat{W}$ for a diamond-shaped interferometer are demonstrated in Section 5. Then the Schrödinger equations at sites u=0,1 read

(3) $$\begin{array}{ccc} y_{0}|\psi (0)\rangle +\hat{W}|\psi (1)\rangle -j|\psi (-1)\rangle & = & \epsilon |\psi (0)\rangle , \end{array}$$

(4) $$\begin{array}{ccc} y_{1}|\psi (1)\rangle +{\hat{W}}^{†}|\psi (0)\rangle -j|\psi (2)\rangle & = & \epsilon |\psi (1)\rangle . \end{array}$$

The reflection and transmission amplitude matrices for the electron flux with an energy $\epsilon =\epsilon_{k}$ injected from the left lead is

(5) $$\begin{array}{ccc} \hat{r} & = & -I+i\eta_{k}X_{1}{\left[IY-\hat{W}{\hat{W}}^{†}\right]}^{-1}, \end{array}$$

(6) $$\begin{array}{ccc} \hat{t} & = & i\eta_{k}{\hat{W}}^{†}{\left[IY-\hat{W}{\hat{W}}^{†}\right]}^{-1}, \end{array}$$

where $Y\equiv X_{0}X_{1}$ with complex parameters $X_{u}\equiv \epsilon_{k}-y_{u}+je^{ika}$ (u=0,1) and we introduced a parameter of energy dimension $\eta_{k}\equiv 2j\sin\left(ka\right)$. The reflection and transmission amplitude matrices for the electron flux injected from the right lead is

(7) $$\begin{array}{ccc} {\hat{r}}^{\prime } & = & -I+i\eta_{k}X_{0}{\left[IY-{\hat{W}}^{†}\hat{W}\right]}^{-1}, \end{array}$$

(8) $$\begin{array}{ccc} {\hat{t}}^{\prime } & = & i\eta_{k}\hat{W}{\left[IY-{\hat{W}}^{†}\hat{W}\right]}^{-1}. \end{array}$$

The details of the derivation of these formulae are given in Appendix A. In the next section, the obtained scattering amplitude matrices are diagonalized and the formulae of the scattering amplitude eigenvalues are given. Then in Section 4, the Berry curvatures for two spin eigenstates, Equations (34) and (35), is given, which allow calculation of QAP spin per cycle.

## 3. Diagonalization of Hopping Operator $\hat{W}{\hat{W}}^{†}$

In this section, we diagonalize the product of hopping operators $\hat{W}$ and ${\hat{W}}^{†}$ appearing in the scattering amplitude matrices derived in the previous section. Then we obtain the scattering eigenstates through an interferometer. This is an extension of the discussion in Reference [3]. We consider an interferometer in x−y plane made of two one-dimensional arms, *b* and *c*, represented by real coupling parameters $\gamma_{b},\gamma_{c}$ and 2×2 unitary matrices, ${\hat{U}}_{b}$ and ${\hat{U}}_{c}$, showing propagation from the site 0 to 1 via the arms *b* and *c*, respectively. We assume following general expressions characterizing the effect of AB phase and Rashba or Dresselhaus SOI:

(9) $$\begin{array}{ccc} {\hat{U}}_{b} & = & e^{-i\phi_{1}}\left(I\delta +i\tau \cdot \hat{\sigma }\right), \end{array}$$

(10) $$\begin{array}{ccc} {\hat{U}}_{c} & = & e^{i\phi_{2}}\left(I\delta^{\prime }+i\tau^{\prime }\cdot \hat{\sigma }\right), \end{array}$$

where $\hat{\sigma }$ is the vector of Pauli spin matrices. $\phi \equiv \phi_{1}+\phi_{2}=2\pi \left(HS\right)/\Phi_{0}$ is the AB phase with the magnetic field *H* in the *z* direction, the area of the interferometer *S*, and a magnetic flux quantum $\Phi_{0}$. Unitarity condition requires the real parameters, $\delta ,\delta^{\prime }$ and real three-dimensional vectors $\tau ,\tau^{\prime }$ to obey $\delta^{2}+{\left|\tau \right|}^{2}=\delta^{\prime 2}+{\left|\tau^{\prime }\right|}^{2}=1$. The hopping matrix $\hat{W}$ is given by

(11) $$\begin{array}{ccc} \hat{W} & = & \gamma_{b}{\hat{U}}_{b}+\gamma_{c}{\hat{U}}_{c}. \end{array}$$

As shown in Appendix B, the matrix factor appearing in the scattering amplitudes for the electron flux injected from the left lead, Equation (5), is

(12) $$\begin{array}{ccc} \hat{W}{\hat{W}}^{†} & \equiv & AI+B\cdot \hat{\sigma }, \end{array}$$

where

(13) $$\begin{array}{ccc} A & = & \gamma_{b}^{2}+\gamma_{c}^{2}+2\gamma_{b}\gamma_{c}\cos\phi \cos\omega , \end{array}$$

(14) $$\begin{array}{ccc} B & = & 2\gamma_{b}\gamma_{c}\sin\phi \sin\omega \;\hat{n}. \end{array}$$

The real parameter ω is determined from $\cos\omega \equiv \delta \delta^{\prime }+\tau \cdot \tau^{\prime }$ and the unit vector $\hat{n}$ is defined by

(15) $$\begin{array}{ccc} \hat{n} & = & \frac{1}{\sqrt{1-\tau_{z}^{2}}}\left(-\tau_{y},\tau_{x},\delta \right). \end{array}$$

We then introduce two normalized eigenstates of the operator $\hat{n}\cdot \hat{\sigma }$, $|\hat{n}\rangle$ and $|-\hat{n}\rangle$ such that

(16) $$\begin{array}{ccc} \hat{n}\cdot \hat{\sigma }|\hat{n}\rangle & = & |\hat{n}\rangle , \end{array}$$

(17) $$\begin{array}{ccc} \hat{n}\cdot \hat{\sigma }|-\hat{n}\rangle & = & -|-\hat{n}\rangle . \end{array}$$

Clearly, these are also the eigenstates of the operator $\hat{W}{\hat{W}}^{†}$ such that

(18) $$\begin{array}{ccc} \hat{W}{\hat{W}}^{†}|\pm \hat{n}\rangle & = & \lambda_{\pm }|\pm \hat{n}\rangle , \end{array}$$

with the eigenvalues

(19) $$\begin{array}{ccc} \lambda_{\pm } & = & \gamma_{b}^{2}+\gamma_{c}^{2}+2\gamma_{b}\gamma_{c}\cos\left(\phi \mp \omega \right). \end{array}$$

These eigenvalues are positive since $\lambda_{\pm }=\langle \pm \hat{n}|\hat{W}{\hat{W}}^{†}|\pm \hat{n}\rangle ={\left|\left|{\hat{W}}^{†}|\pm \hat{n}\rangle \right|\right|}^{2}\geq 0.$

To study the scattering eigenstates for the electron flux injected from the right, Equation (7), we evaluate ${\hat{W}}^{†}\hat{W}$ with similar procedure as above,

(20) $$\begin{array}{ccc} {\hat{W}}^{†}\hat{W} & \equiv & AI+B^{\prime }\cdot \hat{\sigma }, \end{array}$$

where

(21) $$\begin{array}{ccc} B^{\prime } & = & 2\gamma_{b}\gamma_{c}\sin\phi \sin\omega \;{\hat{n}}^{\prime }, \end{array}$$

and corresponding unit vector

(22) $$\begin{array}{ccc} {\hat{n}}^{\prime } & = & \frac{1}{\sqrt{1-\tau_{z}^{2}}}\left(\tau_{y},-\tau_{x},\delta \right). \end{array}$$

Then we introduce two normalized eigenstates of the operator ${\hat{n}}^{\prime }\cdot \hat{\sigma }$, $|\pm {\hat{n}}^{\prime }\rangle$, which obey

(23) $$\begin{array}{ccc} {\hat{W}}^{†}\hat{W}|\pm {\hat{n}}^{\prime }\rangle & = & \lambda_{\pm }|\pm {\hat{n}}^{\prime }\rangle , \end{array}$$

with the same eigenvalues as Equation (18).

The elements of the scattering matrix are now explicitly evaluated with the obtained scattering eigenstates. As detailed in Appendix B, we can show that the transmission amplitude matrices are

(24) $$\begin{array}{ccc} \hat{t} & = & t_{+}|{\hat{n}}^{\prime }\rangle \langle \hat{n}|+t_{-}|-{\hat{n}}^{\prime }\rangle \langle -\hat{n}|, \end{array}$$

(25) $$\begin{array}{ccc} {\hat{t}}^{\prime } & = & t_{+}|\hat{n}\rangle \langle {\hat{n}}^{\prime }|+t_{-}|-\hat{n}\rangle \langle -{\hat{n}}^{\prime }|, \end{array}$$

where we defined two transmission amplitudes,

(26) $$\begin{array}{ccc} t_{\pm } & \equiv & \frac{i\eta_{k}\sqrt{\lambda_{\pm }}}{Y-\lambda_{\pm }}. \end{array}$$

Similarly, the reflection amplitude matrices are given by

(27) $$\begin{array}{ccc} \hat{r} & = & r_{+}|\hat{n}\rangle \langle \hat{n}|+r_{-}|-\hat{n}\rangle \langle -\hat{n}|, \end{array}$$

(28) $$\begin{array}{ccc} {\hat{r}}^{\prime } & = & r_{+}^{\prime }|{\hat{n}}^{\prime }\rangle \langle {\hat{n}}^{\prime }|+r_{-}^{\prime }|-{\hat{n}}^{\prime }\rangle \langle -{\hat{n}}^{\prime }|, \end{array}$$

where the reflection amplitudes are

(29) $$\begin{array}{ccc} r_{\pm } & \equiv & -1+\frac{i\eta_{k}X_{1}}{Y-\lambda_{\pm }}, \end{array}$$

(30) $$\begin{array}{ccc} r_{\pm }^{\prime } & \equiv & -1+\frac{i\eta_{k}X_{0}}{Y-\lambda_{\pm }}. \end{array}$$

The unitarity condition of the scattering amplitude matrices, ${\hat{t}}^{†}\hat{t}+{\hat{r}}^{†}\hat{r}=1$, is confirmed in Appendix C. The unitarity condition ${\hat{t}}^{{}^{\prime }†}{\hat{t}}^{\prime }+{\hat{r}}^{{}^{\prime }†}{\hat{r}}^{\prime }=1$ can also be checked.

## 4. Quantum Adiabatic Pump

For a non-interacting system, the response (particle transfer) to the slow modulation of the system’s controlling parameters is well described by Brouwer’s formula [19], which is expressed by the elements of the scattering matrix. The particles induced in the left lead in one cycle of the adiabatic modulation of two control parameters $g_{1}$ and $g_{2}$ is

(31) $$\begin{array}{ccc} n & = & \sum_{\sigma }n_{\sigma }, \end{array}$$

(32) $$\begin{array}{ccc} n_{\sigma } & = & -\int_{S}dg_{1}dg_{2}\prod_{\sigma }\left(g_{1},g_{2}\right), \end{array}$$

where *S* is the area in the two-dimensional control parameter space whose edge corresponds to the trajectory of the cycle. The Berry curvature $\prod_{\sigma }\left(g_{1},g_{2}\right)$ for spin σ is

(33) $$\begin{array}{ccc} \prod_{\sigma }\left(g_{1},g_{2}\right) & = & \frac{1}{\pi }ℑ\langle \sigma |\left\{\frac{\partial \hat{r}}{\partial g_{2}}\frac{\partial {\hat{r}}^{†}}{\partial g_{1}}+\frac{\partial \hat{t^{\prime }}}{\partial g_{2}}\frac{\partial {\hat{t^{\prime }}}^{†}}{\partial g_{1}}\right\}|\sigma \rangle , \end{array}$$

where $\hat{r}$ and ${\hat{t}}^{\prime }$ are given in Equations (27) and (25) and |σ〉 is the spinor vector of spin σ.

If we choose the AB phase ϕ and parameters of the interferometers, for example, $X_{0}$ or $X_{1}$, but not the SOI strengths, we can show that the Berry curvature is finite in general as studied in Reference [26]. In the following, however, we focus on the situation that the control parameters are the AB phase ϕ and Rashba or Dresselhaus SOI strength that modulate the eigenvalue $\lambda_{\pm }$ as well as the scattering eigenstates $|\pm \hat{n}\rangle ,|\pm {\hat{n}}^{\prime }\rangle$. To calculate the Berry curvature, we need to evaluate the derivatives of the scattering amplitude matrices, $\hat{r}$ and ${\hat{t}}^{\prime }$. Then, as shown in Appendix D, after some manipulations, we have the Berry curvatures for spin components parallel to $\pm \hat{n}$,

(34) $$\begin{array}{ccc} \prod_{\hat{n}}\left(g_{1},g_{2}\right) & = & \left({\left|r_{+}-r_{-}\right|}^{2}-{\left|t_{+}\right|}^{2}+{\left|t_{-}\right|}^{2}\right)C_{g_{1},g_{2}}, \end{array}$$

and

(35) $$\begin{array}{ccc} \prod_{-\hat{n}}\left(g_{1},g_{2}\right) & = & \left(-{\left|r_{+}-r_{-}\right|}^{2}-{\left|t_{+}\right|}^{2}+{\left|t_{-}\right|}^{2}\right)C_{g_{1},g_{2}}. \end{array}$$

where the factor at the end is independent of spin and is defined as

(36) $$\begin{array}{lll} C_{g_{1},g_{2}} & = & \frac{1}{4\pi n_{z}}\frac{1}{1-\tau_{z}^{2}}[\frac{\partial \tau_{y}}{\partial g_{1}}\frac{\partial \tau_{x}}{\partial g_{2}}-\frac{\partial \tau_{y}}{\partial g_{2}}\frac{\partial \tau_{x}}{\partial g_{1}}\\ & + & \frac{\tau_{z}}{1-\tau_{z}^{2}}\left\{\tau_{x}\left(\frac{\partial \tau_{y}}{\partial g_{1}}\frac{\partial \tau_{z}}{\partial g_{2}}-\frac{\partial \tau_{y}}{\partial g_{2}}\frac{\partial \tau_{z}}{\partial g_{1}}\right)+\tau_{y}\left(\frac{\partial \tau_{x}}{\partial g_{2}}\frac{\partial \tau_{z}}{\partial g_{1}}-\frac{\partial \tau_{x}}{\partial g_{1}}\frac{\partial \tau_{z}}{\partial g_{2}}\right)\right\}]. \end{array}$$

This is one of the main results of this work.

The vector τ is independent of ϕ, but only depends on the SOI strength. Therefore, when one chose the AB phase, $g_{1}\equiv \phi$, as one of the control parameters, $C_{\phi ,g_{2}}$ is identically zero as is evident from Equation (36). Hence we do not expect QAP by modulating the AB phase and SOI strength. It is also obvious that if we chose $\gamma_{b}$ or $\gamma_{c}$ as one of the control parameters and the other by SOI strength, $C_{\gamma_{b,c},g_{2}}$ is zero since τ is independent of $\gamma_{b}$ and $\gamma_{c}$ and no pumping is expected.

Even for a fixed AB phase, there is still some freedom to choose two control parameters related to the SOI strength since we have two types of SOI interaction mechanisms, Rashba and Dresselhaus SOI. In the next section, we study Rashba-Dresselhaus interferometer in a simple diamond-shape structure made of four sites and choose the strengths of two types of SOI as control parameters.

## 5. Diamond Interferometer

We consider an electron transport in two-dimensional system on [001] surface with setting *x* and *y* axis along the (100) and (010) crystal directions, respectively. The Hamiltonian for the SOI is

(37) $$\begin{array}{ccc} {\hat{H}}_{R} & = & \frac{\hbar }{m}k_{R}\left({\hat{p}}_{y}{\hat{\sigma }}_{x}-{\hat{p}}_{x}{\hat{\sigma }}_{y}\right), \end{array}$$

(38) $$\begin{array}{ccc} {\hat{H}}_{D} & = & \frac{\hbar }{m}k_{D}\left({\hat{p}}_{x}{\hat{\sigma }}_{x}-{\hat{p}}_{y}{\hat{\sigma }}_{y}\right), \end{array}$$

where $k_{R}$ and $k_{D}$ are Rashba and Dresselhaus parameters, respectively. ${\hat{p}}_{\mu }$ (μ=x,y) are the momentum and *m* is the electron effective mass.

**Figure 2:**
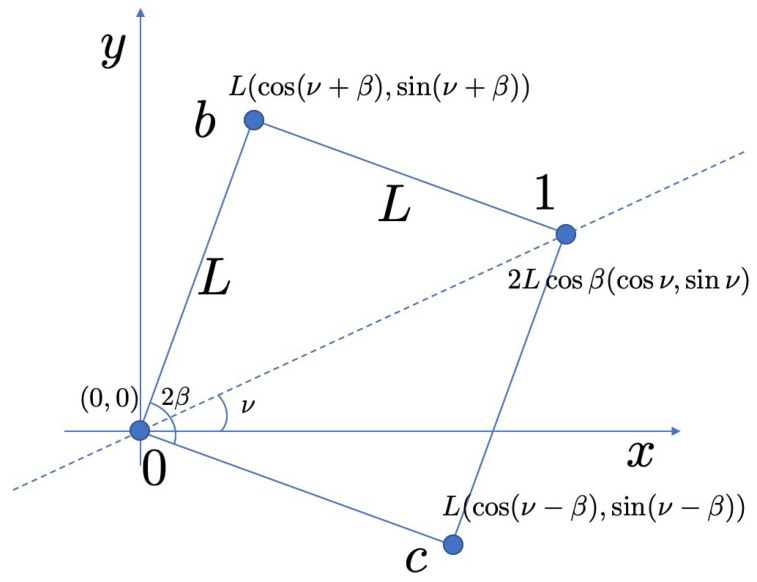
Schematics of the interferometer made of four sites, 0,b,c, and 1 separated by a length *L*. The opening angle 2β and relative angle ν from *x* axis determine the geometric structure.

The interferometer made of four sites is configured as in Figure 2 which is attached to the leads at site u=0 and u=1 as discussed in Reference [3]. Other two sites constituting the interferometer are u=b and u=c, connected with bonds of length *L*. We also define the opening angle 2β and the relative angle ν of the diagonal line to *x* axis. The Hamiltonian reads

(39) $$\begin{array}{ccc} {\hat{H}}_{\mathrm{IF}} & \equiv & \sum_{u}\epsilon_{u}|\psi (u)\rangle \langle \psi (u)|-\sum_{uv}{\tilde{U}}_{uv}|\psi (v)\rangle \langle \psi (u)|, \end{array}$$

for u,v=0,c,d,1 where $\epsilon_{u}$ is the site energy and ${\tilde{U}}_{uv}\equiv J_{uv}{\hat{U}}_{uv}$, $J_{uv}$ is a hopping energy and ${\hat{U}}_{uv}$ is a 2×2 unitary matrix representing the effect of SOI and AB phase. Total Hamiltonian is $\hat{H}={\hat{H}}_{\mathrm{IF}}+{\hat{H}}_{L}+{\hat{H}}_{R}$. In the Appendix E, we explain how this problem is reduced to the Schrödinger equations, Equations (3) and (4).

The coordinates of the four sites are $r_{0}=\left(0,0\right)$, $r_{b}=\left(L\cos\left(\nu +\beta \right),L\sin\left(\nu +\beta \right)\right)$, $r_{1}=\left(2L\cos\left(\beta \right)\cos\left(\nu \right),2L\cos\left(\beta \right)\sin\left(\nu \right)\right)$, and $r_{c}=\left(L\cos\left(\nu -\beta \right),L\sin\left(\nu -\beta \right)\right)$. We define $\alpha_{R}\equiv k_{R}L$, $\alpha_{D}\equiv k_{D}L$ and $\zeta \equiv \sqrt{\alpha_{R}^{2}+\alpha_{D}^{2}}$ and introduce another angle θ, such that $\alpha_{R}=\zeta \cos\theta$, and $\alpha_{D}=\zeta \sin\theta$. The unitary matrix for the hopping from site at (0,0) to site at $\left(u_{x},u_{y}\right)$ is ${\hat{U}}_{\left(0,0\right),\left(u_{x},u_{y}\right)}=\exp\left[iK\cdot \hat{\sigma }\right]$ with $K\equiv \alpha_{R}\left(u_{y},-u_{x},0\right)+\alpha_{D}\left(u_{x},-u_{y},0\right)$ [27]. Therefore, for the hopping from site 0 to *b*,

(40) $$\begin{array}{ccc} K_{0b}\cdot \hat{\sigma } & = & \zeta \sin\left(\xi_{1}\right){\hat{\sigma }}_{x}-\zeta \cos\left(\xi_{2}\right){\hat{\sigma }}_{y}\equiv \zeta {\hat{\sigma }}_{1}, \end{array}$$

with $\xi_{1}\equiv \beta +\nu +\theta$ and $\xi_{2}\equiv \beta +\nu -\theta$. Similarly, for the hopping from site *c* to 0,

(41) $$\begin{array}{ccc} K_{c0}\cdot \hat{\sigma } & = & \zeta \sin\left(\xi_{4}\right){\hat{\sigma }}_{x}+\zeta \cos\left(\xi_{3}\right){\hat{\sigma }}_{y}\equiv \zeta {\hat{\sigma }}_{2}, \end{array}$$

with $\xi_{3}\equiv \beta -\nu +\theta$ and $\xi_{4}\equiv \beta -\nu -\theta$. We introduce factors $F_{1}\equiv \sqrt{1+\sin(2\nu +2\beta )\sin(2\theta )}$ and $F_{2}\equiv \sqrt{1+\sin(2\nu -2\beta )\sin(2\theta )}$ such that ${\hat{\sigma }}_{1}^{2}=IF_{1}^{2}$ and ${\hat{\sigma }}_{2}^{2}=IF_{2}^{2}$. Then, for n=1,2,

(42) $$\begin{array}{ccc} e^{i\zeta {\hat{\sigma }}_{n}} & \equiv & Ic_{n}+is_{n}{\hat{\sigma }}_{n}, \end{array}$$

where we defined

(43) $$\begin{array}{cc} c_{n}\equiv \cos\left(F_{n}\zeta \right),\;\; & \;s_{n}\equiv \frac{1}{F_{n}}\sin\left(F_{n}\zeta \right). \end{array}$$

Noting that ${\hat{U}}_{0b}=e^{i\zeta {\hat{\sigma }}_{1}}$, ${\hat{U}}_{b1}=e^{-\frac{i\phi }{2}-i\zeta {\hat{\sigma }}_{2}}$, ${\hat{U}}_{0c}=e^{-i\zeta {\hat{\sigma }}_{2}}$ and ${\hat{U}}_{c1}=e^{\frac{i\phi }{2}+i\zeta {\hat{\sigma }}_{1}}$,

(44) $$\begin{array}{lll} {\hat{U}}_{b} & \equiv & {\hat{U}}_{0b}{\hat{U}}_{b1}\\ & = & e^{-\frac{i\phi }{2}}\left\{Ic_{1}c_{2}-ic_{1}s_{2}{\hat{\sigma }}_{2}+ic_{2}s_{1}{\hat{\sigma }}_{1}+s_{1}s_{2}{\hat{\sigma }}_{1}{\hat{\sigma }}_{2}\right\}\\ & = & e^{-\frac{i\phi }{2}}\left(I\delta +i\tau \cdot \hat{\sigma }\right), \end{array}$$

where

(45) $$\begin{array}{ccc} \delta & \equiv & c_{1}c_{2}-s_{1}s_{2}\left(\sin\left(2\nu \right)\sin\left(2\theta \right)+\cos\left(2\beta \right)\right),\\ \tau_{x} & \equiv & -c_{1}s_{2}\sin\xi_{4}+c_{2}s_{1}\sin\xi_{1},\\ \tau_{y} & \equiv & -c_{1}s_{2}\cos\xi_{3}-c_{2}s_{1}\cos\xi_{2},\\ \tau_{z} & \equiv & s_{1}s_{2}\sin\left(2\beta \right)\cos\left(2\theta \right). \end{array}$$

Similarly,

(46) $$\begin{array}{lll} {\hat{U}}_{c} & \equiv & {\hat{U}}_{0c}{\hat{U}}_{c1}\\ & = & e^{\frac{i\phi }{2}}\left\{Ic_{2}c_{1}-is_{2}c_{1}{\hat{\sigma }}_{2}+ic_{2}s_{1}{\hat{\sigma }}_{1}+s_{2}s_{1}{\hat{\sigma }}_{2}{\hat{\sigma }}_{1}\right\}\\ & = & e^{\frac{i\phi }{2}}\left(I\delta^{\prime }+i\tau^{\prime }\cdot \hat{\sigma }\right), \end{array}$$

with $\delta^{\prime }=\delta$ and $\tau^{\prime }=\left(\tau_{x},\tau_{y},-\tau_{z}\right)$. The angle ω is determined by $\cos\omega =\delta \delta^{\prime }+\tau \cdot \tau^{\prime }=\delta^{2}+\tau_{x}^{2}+\tau_{y}^{2}-\tau_{z}^{2}=1-2\tau_{z}^{2}$.

## 6. QAP in the Diamond Interferometer

We examine the quantum adiabatic spin-pumping by choosing two SOI strengths $g_{1}=\alpha_{R}$ and $g_{2}=\alpha_{D}$ as control parameters. First we examine the basic property of the function $C_{\alpha_{R},\alpha_{D}}$ defined in Equation (36) and then evaluate the scattering amplitudes. Using these results, we calculate the Berry curvatures for two spin directions.

### 6.1. Spin-Independent Function $C_{\alpha_{R},\alpha_{D}}$

The function $C_{\alpha_{R},\alpha_{D}}$ has symmetries, $C_{\alpha_{1},\alpha_{2}}=C_{\alpha_{2},\alpha_{1}}$, as well as $C_{\alpha_{1},\alpha_{2}}=C_{-\alpha_{1},\alpha_{2}},C_{\alpha_{1},\alpha_{2}}=C_{\alpha_{1},-\alpha_{2}}.$ Moreover, it also obeys the relation ${\left.C_{\alpha_{R},\alpha_{D}}\right|}_{\nu }=-{\left.C_{\alpha_{R},\alpha_{D}}\right|}_{\frac{\pi }{2}-\nu }$. Therefore, the angle ν=π/4 is rather special. At this angle, $C_{\alpha_{R},\alpha_{D}}$ is identically zero and hence no pumping. One can check this since $F_{1}=F_{2}$ and hence $c_{1}=c_{2}$ and $s_{1}=s_{2}$, then

(47) $$\begin{array}{ccc} \tau_{x} & = & c_{1}s_{1}\left(\sin\xi_{1}-\sin\xi_{4}\right)=2c_{1}s_{1}\cos\beta \sin\left(\theta +\frac{\pi }{4}\right), \end{array}$$

(48) $$\begin{array}{ccc} \tau_{y} & = & -c_{1}s_{1}\left(\cos\xi_{3}+\cos\xi_{2}\right)=-2c_{1}s_{1}\cos\beta \cos\left(\theta -\frac{\pi }{4}\right). \end{array}$$

Therefore, the relation $\tau_{x}=-\tau_{y}$ holds for any β and θ and $C_{\alpha_{R},\alpha_{D}}=0$.

**Figure 3:**
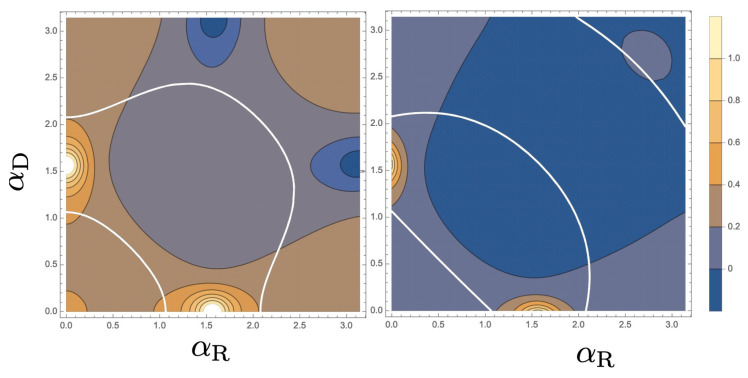
Contour plot of the function $C_{\alpha_{R},\alpha_{D}}$ depending on the Rashba, $\alpha_{R}$, and Dresselhaus, $\alpha_{D}$, SOI strength parameters. We chose the geometric angles β=π/5 and ν=π/2 (**left**) and ν=3π/8 (**right**).

Because of its symmetric property, we focus on the function $C_{\alpha_{R},\alpha_{D}}$ in the range $0\leq \alpha_{R},\alpha_{D}\leq \pi$. As an example, we chose β=π/5 and the results for ν=π/2 and ν=3π/8 are shown in Figure 3. The result for ν=π/4 is uniformly zero as noted above and that for ν=π/8 is similar to that for ν=3π/8 with reversing the sign of the function. There are areas where the absolute value of $C_{\alpha_{R},\alpha_{D}}$ is enhanced near $\left(\alpha_{R},\alpha_{D}\right)=\left(\frac{\pi }{2},0\right),\left(0,\frac{\pi }{2}\right)$, which can be understood from Equation (36) since $\left|\tau_{z}\right|$ is very close to one. If we choose β=π/4, the scattering states “flips” at π/2 when $\alpha_{R}$ is increased from zero to π with $\alpha_{D}=0$ [3]. Then the behavior of $C_{\alpha_{R},\alpha_{D}=0}$ becomes quite singular, which may need further investigation (not being discussed here).

### 6.2. Spin-Dependent Prefactors

**Figure 4:**
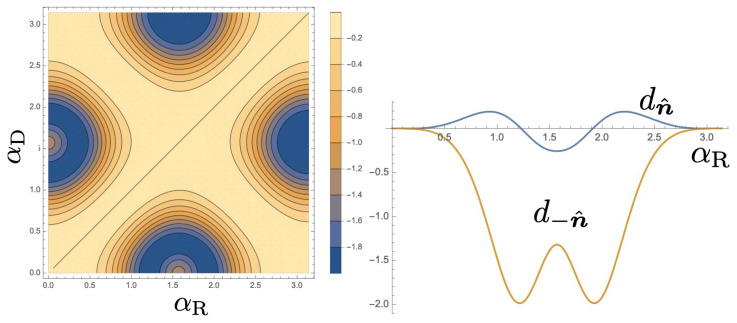
(**Left**) Contour plot of the function $d_{-\hat{n}}$ for $\epsilon_{0}=0.9j$ and ϕ=π/3. (**Right**) Line plot of the functions $d_{\pm \hat{n}}$ as a function of $\alpha_{R}$ with $\alpha_{D}=0$.

**Figure 5:**
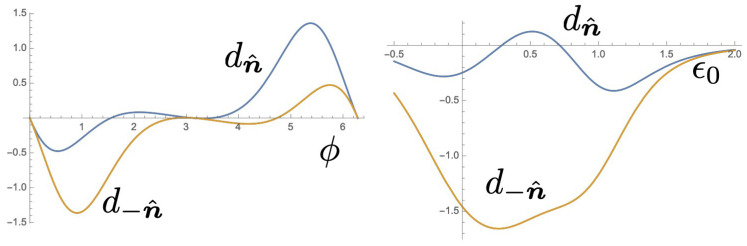
(**Left**) AB phase dependence of the function $d_{\pm \hat{n}}$ for $\epsilon_{0}=0.9j$ and $\alpha_{R}=\pi /2,\alpha_{D}=0$. (**Right**) Site energy dependence of the function $d_{\pm \hat{n}}$ for f=π/3 and $\alpha_{R}=\pi /2,\alpha_{D}=0$.

In this section, we examine the scattering amplitudes, $t_{\pm }$ and $r_{\pm }$ and the prefactors of the Berry curvatures in Equations (34) and (35). We define these factors as $d_{\hat{n}}={\left|r_{+}-r_{-}\right|}^{2}-{\left|t_{+}\right|}^{2}+{\left|t_{-}\right|}^{2}$ and $d_{-\hat{n}}=-{\left|r_{+}-r_{-}\right|}^{2}-{\left|t_{+}\right|}^{2}+{\left|t_{-}\right|}^{2}$. To be compatible with the analysis in the previous subsection, we focus on the geometry such that β=π/5 and ν=π/2. For simplicity, we chose symmetric setup of the interferometer, where $J_{0b}=J_{b1}=J_{0c}=J_{c1}=j$ and $\epsilon_{0}=\epsilon_{1}=\epsilon_{b}=\epsilon_{c}$. Then, $\gamma_{b}=\gamma_{c}=\frac{j^{2}}{\epsilon_{k}-\epsilon_{0}}$. Moreover, in the following calculation we chose $\epsilon_{k}=-j$. First, we show the result of $d_{-\hat{n}}$ for $\epsilon_{0}=0.9j$ in Figure 4 with choosing the AB phase ϕ=π/3. This function is negatively enhanced near $\left(\alpha_{R},\alpha_{D}\right)=\left(\pi /2,0\right)$ and (0,π/2). In contrast, the factor $d_{\hat{n}}$ is much smaller as shown in the linear plot for $\alpha_{D}=0$. If one chose AB phase ϕ=5π/3, $d_{-\hat{n}}$ is suppressed and alternatively $d_{\hat{n}}$ is enhanced near $\left(\alpha_{R},\alpha_{D}\right)=\left(\pi /2,0\right)$ and (0,π/2) (with changing sign of the data in the left Figure 4). The AB phase ϕ and site energy $\epsilon_{0}$ dependence of $d_{\pm \hat{n}}$ are shown in the left and right of Figure 5, respectively. Therefore, a large contrast of the QAP in two spin directions can be obtained by choosing ϕ=π/3 and $\epsilon_{0}=0.9j$.

### 6.3. Berry Curvatures

**Figure 6:**
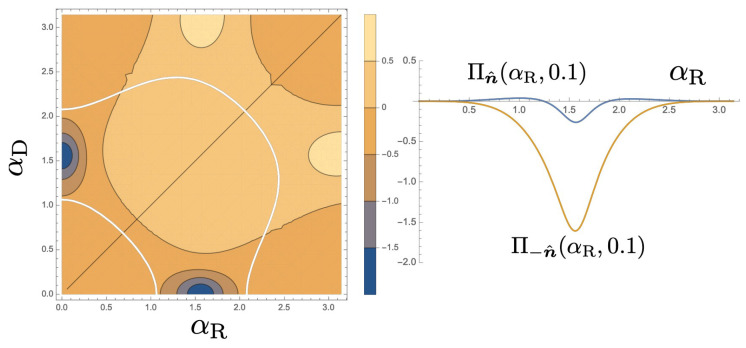
(**Left**) Contour plot of the Berry curvature for $|-\hat{n}\rangle$ with β=π/5 and ν=π/2. We set ϕ=π/3, $\epsilon_{k}=-j$, $J_{0b}=J_{b1}=J_{0c}=J_{c1}=j$ and $\epsilon_{0}=\epsilon_{1}=\epsilon_{b}=\epsilon_{c}=0.9j$. (**Right**) Berry curvatures for two spin directions with $\alpha_{D}=0.1$ with the same parameter with the left panel.

Finally, we calculate the Berry curvature, $\prod_{-\hat{n}}\left(\alpha_{R},\alpha_{D}\right)$ for the spin in the state $|-\hat{n}\rangle$ as shown in the left of Figure 6. Obviously, the Berry curvature becomes large at around $\left(\alpha_{R},\alpha_{D}\right)=\left(\frac{\pi }{2},0\right),\left(0,\frac{\pi }{2}\right)$. The other spin state is not much pumped as shown in the right of Figure 6.

## 7. Discussion

We have derived a general expression of the Berry curvature for an interferometer connected to one-dimensional leads. In this study, we restricted the control parameters in QAP formalism only to modulate the scattering eigenstates and corresponding eigenvalues through the change of the unitary operators for each arm. Then the AB phase, which, despite modifying the scattering eigenvalues, $\lambda_{\pm }$, does not affect the scattering eigenstates and is shown not to function as a control parameter in QAP. In a clear contrast, it has been shown [26] that in combination with the potential modulation, affecting the electron-hopping amplitudes or site energies, QAP by AB phase is possible.

In the current analysis, the control parameters are assumed to purely modulate the phase of the electrons. In real experiments, unintended modulation of hopping amplitudes, $J_{uv}$, or the site energies, $\epsilon_{b}$ or $\epsilon_{c}$ by the gate voltages may induce additional effects. We demonstrated that by using the two types of the SOI as the two control parameters, spin-QAP is possible. However, in the experiments, independent control of the Rashba SOI and Dresselhaus SOI will be a complicated task. Fortunately, as shown in Figure 6, the area of large Berry curvature is well isolated and the tiny change of Dresselhaus SOI may be sufficient to observe QAP. It would be interesting if other types of SOI interaction [5] could be another control parameter of the QAP.

## Abbreviations

The following abbreviations are used in this manuscript:

SOI	Spin-orbit interaction
QAP	Quantum adiabatic pumping
AB	Aharonov-Bohm

## Appendix A. Scattering Matrix

In this Appendix, we argue the scattering problem through the interferometer. First, we inject an electron flux with an energy $\epsilon =\epsilon_{k}$ from the left lead. The wavefunction for u≤0 is

(A1) $$\begin{array}{ccc} |\psi (u)\rangle & = & e^{ikua}|\chi_{\mathrm{in}}\rangle +e^{-ikua}|\chi_{r}\rangle , \end{array}$$

and the wavefunction for u≥1 is

(A2) $$\begin{array}{ccc} |\psi (u)\rangle & = & e^{ik(u-1)a}|\chi_{t}\rangle , \end{array}$$

where $|\chi_{\mathrm{in}}\rangle$ is the injected wavefunction and $|\chi_{r}\rangle ,|\chi_{t}\rangle$ are the (un-normalized) wavefunctions of reflection and transmission. In particular, at sites u=0,−1,

(A3) $$\begin{array}{cc} |\psi (0)\rangle =|\chi_{\mathrm{in}}\rangle +|\chi_{r}\rangle ,\;\; & \;\;|\psi (-1)\rangle =e^{-ika}|\chi_{\mathrm{in}}\rangle +e^{ika}|\chi_{r}\rangle , \end{array}$$

and at sites u=1,2,

(A4) $$\begin{array}{cc} |\psi (1)\rangle =|\chi_{t}\rangle ,\;\; & \;\;|\psi (2)\rangle =e^{ika}|\chi_{t}\rangle . \end{array}$$

By putting these into Equations (3) and (4), we have

(A5) $$\begin{array}{ccc} \left(\epsilon_{k}-y_{0}\right)\left\{|\chi_{\mathrm{in}}\rangle +|\chi_{r}\rangle \right\} & = & \hat{W}|\chi_{t}\rangle -j\left\{e^{-ika}|\chi_{\mathrm{in}}\rangle +e^{ika}|\chi_{r}\rangle \right\}, \end{array}$$

(A6) $$\begin{array}{ccc} (\epsilon_{k}-y_{1})|\chi_{t}\rangle & = & {\hat{W}}^{†}\left\{|\chi_{\mathrm{in}}\rangle +|\chi_{r}\rangle \right\}-je^{ika}|\chi_{t}\rangle . \end{array}$$

Then from Equation (A6),

(A7) $$\begin{array}{ccc} |\chi_{t}\rangle & = & \frac{1}{\epsilon_{k}-y_{1}+je^{ika}}{\hat{W}}^{†}\left\{|\chi_{\mathrm{in}}\rangle +|\chi_{r}\rangle \right\}, \end{array}$$

and putting this into Equation (A5), we have

(A8) $$\begin{array}{c} \left(\epsilon_{k}-y_{0}+je^{-ika}\right)|\chi_{\mathrm{in}}\rangle +\left(\epsilon_{k}-y_{0}+je^{ika}\right)|\chi_{r}\rangle =\hat{W}\frac{1}{\epsilon_{k}-y_{1}+je^{ika}}{\hat{W}}^{†}\left\{|\chi_{\mathrm{in}}\rangle +|\chi_{r}\rangle \right\}. \end{array}$$

Defining complex parameters $X_{u}\equiv \epsilon_{k}-y_{u}+je^{ika}$ (u=0,1) and noting $\epsilon_{k}-y_{0}+je^{-ika}=X_{0}-i\eta_{k}$, we solve this equation

$$\begin{array}{ccc} |\chi_{r}\rangle & = & -{\left[IX_{0}X_{1}-\hat{W}{\hat{W}}^{†}\right]}^{-1}\left[IX_{0}X_{1}-\hat{W}{\hat{W}}^{†}-Ii\eta_{k}X_{1}\right]|\chi_{\mathrm{in}}\rangle . \end{array}$$

Then we have obtained the reflection amplitude matrix

(A9) $$\begin{array}{lll} \hat{r} & \equiv & -{\left[IX_{0}X_{1}-\hat{W}{\hat{W}}^{†}\right]}^{-1}\left[IX_{0}X_{1}-\hat{W}{\hat{W}}^{†}-Ii\eta_{k}X_{1}\right]\\ & = & -I+i\eta_{k}X_{1}{\left[IY-\hat{W}{\hat{W}}^{†}\right]}^{-1}, \end{array}$$

where we have introduced $Y\equiv X_{0}X_{1}$. Using this, transmitted state is calculated with Equation (A7),

$$\begin{array}{lll} |\chi_{t}\rangle & = & \frac{1}{X_{1}}{\hat{W}}^{†}\left\{|\chi_{\mathrm{in}}\rangle +|\chi_{r}\rangle \right\}\\ & = & \frac{1}{X_{1}}{\hat{W}}^{†}\left[I-I+i\eta_{k}X_{1}{\left[IY-\hat{W}{\hat{W}}^{†}\right]}^{-1}\right]|\chi_{\mathrm{in}}\rangle \\ & = & i\eta_{k}{\hat{W}}^{†}{\left[IY-\hat{W}{\hat{W}}^{†}\right]}^{-1}|\chi_{\mathrm{in}}\rangle , \end{array}$$

hence the transmission amplitude matrix is

(A10) $$\begin{array}{ccc} \hat{t} & = & i\eta_{k}{\hat{W}}^{†}{\left[IY-\hat{W}{\hat{W}}^{†}\right]}^{-1}. \end{array}$$

We alternatively consider the situation that the electron is injected from the right lead. The wavefunction for u≥1 is

(A11) $$\begin{array}{ccc} |\psi (n)\rangle & = & e^{-ik(n-1)a}|\chi_{\mathrm{in}}^{\prime }\rangle +e^{ik(n-1)a}|\chi_{r}^{\prime }\rangle , \end{array}$$

and the wavefunction for u≤0 is

(A12) $$\begin{array}{ccc} |\psi (n)\rangle & = & e^{-ikna}|\chi_{t}^{\prime }\rangle , \end{array}$$

where $|\chi_{\mathrm{in}}^{\prime }\rangle$ is the incoming wavefunction and $|\chi_{r}^{\prime }\rangle ,|\chi_{t}^{\prime }\rangle$ are the (un-normalized) wavefunctions of reflection and transmission. At sites u=1,2,

(A13) $$\begin{array}{cc} |\psi (1)\rangle =|\chi_{\mathrm{in}}^{\prime }\rangle +|\chi_{r}^{\prime }\rangle ,\;\; & \;\;|\psi (2)\rangle =e^{-ika}|\chi_{\mathrm{in}}^{\prime }\rangle +e^{ika}|\chi_{r}^{\prime }\rangle , \end{array}$$

and at sites u=0,−1,

(A14) $$\begin{array}{cc} |\psi (0)\rangle =|\chi_{t}^{\prime }\rangle ,\;\; & \;\;|\psi (-1)\rangle =e^{ika}|\chi_{t}^{\prime }\rangle . \end{array}$$

By putting these into Equations (3) and (4), we have

(A15) $$\begin{array}{ccc} (\epsilon_{k}-y_{0})|\chi_{t}^{\prime }\rangle & = & \hat{W}\left\{|\chi_{\mathrm{in}}^{\prime }\rangle +|\chi_{r}^{\prime }\rangle \right\}-je^{ika}|\chi_{t}^{\prime }\rangle , \end{array}$$

(A16) $$\begin{array}{ccc} \left(\epsilon_{k}-y_{1}\right)\left\{|\chi_{\mathrm{in}}^{\prime }\rangle +|\chi_{r}^{\prime }\rangle \right\} & = & {\hat{W}}^{†}|\chi_{t}^{\prime }\rangle -j\left\{e^{-ika}|\chi_{\mathrm{in}}^{\prime }\rangle +e^{ika}|\chi_{r}^{\prime }\rangle \right\}. \end{array}$$

From Equation (A15),

(A17) $$\begin{array}{ccc} |\chi_{t}^{\prime }\rangle & = & \frac{1}{X_{0}}\hat{W}\left\{|\chi_{\mathrm{in}}^{\prime }\rangle +|\chi_{r}^{\prime }\rangle \right\}, \end{array}$$

and putting this into Equation (A16),

(A18) $$\begin{array}{ccc} \left(X_{1}-i\eta_{k}\right)|\chi_{\mathrm{in}}^{\prime }\rangle +X_{1}|\chi_{r}^{\prime }\rangle & = & {\hat{W}}^{†}\frac{1}{X_{0}}\hat{W}\left\{|\chi_{\mathrm{in}}^{\prime }\rangle +|\chi_{r}^{\prime }\rangle \right\}, \end{array}$$

which is solved as

$$\begin{array}{lll} |\chi_{r}^{\prime }\rangle & = & {\left[IY-{\hat{W}}^{†}\hat{W}\right]}^{-1}\left\{-\left(IY-{\hat{W}}^{†}\hat{W}\right)+i\eta_{k}X_{0}I\right\}|\chi_{\mathrm{in}}^{\prime }\rangle \\ & = & \left\{-I+i\eta_{k}X_{0}{\left[IY-{\hat{W}}^{†}\hat{W}\right]}^{-1}\right\}|\chi_{\mathrm{in}}^{\prime }\rangle , \end{array}$$

Therefore, the reflection amplitude matrix is

(A19) $$\begin{array}{ccc} {\hat{r}}^{\prime } & = & -I+i\eta_{k}X_{0}{\left[IY-{\hat{W}}^{†}\hat{W}\right]}^{-1}. \end{array}$$

Putting this into Equation (A17),

$$\begin{array}{lll} |\chi_{t}^{\prime }\rangle & = & \frac{\hat{W}}{X_{0}}\left\{I-I+i\eta_{k}X_{0}{\left[IY-{\hat{W}}^{†}\hat{W}\right]}^{-1}\right\}|\chi_{\mathrm{in}}^{\prime }\rangle \\ & = & i\eta_{k}\hat{W}{\left[IY-{\hat{W}}^{†}\hat{W}\right]}^{-1}|\chi_{\mathrm{in}}^{\prime }\rangle , \end{array}$$

and hence the transmission amplitude matrix is

(A20) $$\begin{array}{ccc} {\hat{t}}^{\prime } & = & i\eta_{k}\hat{W}{\left[IY-{\hat{W}}^{†}\hat{W}\right]}^{-1}. \end{array}$$

## Appendix B. Scattering Eigenstates

In this appendix, we show the details of the calculations of the scattering eigenstates discussed in Section 3. First, we evaluate

(A21) $$\begin{array}{lll} \hat{W}{\hat{W}}^{†} & = & \left(\gamma_{b}{\hat{U}}_{b}+\gamma_{c}{\hat{U}}_{c}\right)\left(\gamma_{b}{\hat{U}}_{b}^{†}+\gamma_{c}{\hat{U}}_{c}^{†}\right)\\ & = & \left(\gamma_{b}^{2}+\gamma_{c}^{2}\right)I+\gamma_{b}\gamma_{c}\left(\hat{u}+{\hat{u}}^{†}\right), \end{array}$$

with $\hat{u}\equiv {\hat{U}}_{b}{\hat{U}}_{c}^{†}$ representing the total development around the interferometer in the order 0→b→1→c→0. We study following matrix

$$\begin{array}{lll} \hat{u} & = & e^{-i\phi }\left(I\delta +i\tau \cdot \hat{\sigma }\right)\left(I\delta^{\prime }-i\tau^{\prime }\cdot \hat{\sigma }\right)\\ & = & e^{-i\phi }\left\{I\delta \delta^{\prime }-i\delta \tau^{\prime }\cdot \hat{\sigma }+i\delta^{\prime }\tau \cdot \hat{\sigma }+\left(\tau \cdot \hat{\sigma }\right)\left(\tau^{\prime }\cdot \hat{\sigma }\right)\right\}\\ & = & e^{-i\phi }\left\{I\delta \delta^{\prime }+i\left(\delta^{\prime }\tau -\delta \tau^{\prime }\right)\cdot \hat{\sigma }+I\tau \cdot \tau^{\prime }+i\left(\tau \times \tau^{\prime }\right)\cdot \hat{\sigma }\right\}. \end{array}$$

We introduce a unit vector defined by

(A22) $$\begin{array}{ccc} \hat{n} & \equiv & N\left\{\delta^{\prime }\tau -\delta \tau^{\prime }+\tau \times \tau^{\prime }\right\}=\left(n_{x},n_{y},n_{z}\right), \end{array}$$

where N is a normalization constant. We define *z*-direction (in spin space) in parallel to $\delta^{\prime }\tau -\delta \tau^{\prime }$, namely

(A23) $$\begin{array}{ccc} N\left\{\delta^{\prime }\tau -\delta \tau^{\prime }\right\} & \equiv & n_{z}\hat{z}, \end{array}$$

ith a unit vector $\hat{z}$ in *z*-direction. Since $\tau \times \tau^{\prime }$ is orthogonal to $\delta^{\prime }\tau -\delta \tau^{\prime }$, we set

(A24) $$\begin{array}{ccc} N\left\{\tau \times \tau^{\prime }\right\} & \equiv & n_{x}\hat{x}+n_{y}\hat{y}, \end{array}$$

with unit vectors in x,y-directions. Solving Equation (A23) for $\tau^{\prime }$, we have

(A25) $$\begin{array}{ccc} \tau^{\prime } & = & \frac{\delta^{\prime }}{\delta }\tau -\frac{n_{z}}{\delta N}\hat{z}, \end{array}$$

and hence

(A26) $$\begin{array}{ccc} \tau \times \tau^{\prime } & = & -\frac{n_{z}}{\delta N}\left(\tau \times \hat{z}\right). \end{array}$$

Using this, we obtain for $\tau =(\tau_{x},\tau_{y},\tau_{z})$,

(A27) $$\begin{array}{ccc} n_{x} & = & \hat{x}\cdot N\left\{\tau \times \tau^{\prime }\right\}=-\frac{n_{z}}{\delta }\tau_{y}. \end{array}$$

Similarly, we also have $n_{y}=\frac{n_{z}}{\delta }\tau_{x}.$ Normalization condition requires

(A28) $$\begin{array}{ccc} 1 & = & {\left(-\frac{n_{z}}{\delta }\tau_{y}\right)}^{2}+{\left(\frac{n_{z}}{\delta }\tau_{x}\right)}^{2}+n_{z}^{2}=\frac{1-\tau_{z}^{2}}{\delta^{2}}n_{z}^{2}, \end{array}$$

hence we determine

(A29) $$\begin{array}{ccc} n_{z} & = & \frac{\delta }{\sqrt{1-\tau_{z}^{2}}}, \end{array}$$

and

(A30) $$\begin{array}{ccc} \hat{n} & = & \frac{1}{\sqrt{1-\tau_{z}^{2}}}\left(-\tau_{y},\tau_{x},\delta \right). \end{array}$$

Therefore,

(A31) $$\begin{array}{lll} \hat{u} & = & e^{-i\phi }\left\{I\left(\delta \delta^{\prime }+\tau \cdot \tau^{\prime }\right)+i\frac{1}{N}\hat{n}\cdot \hat{\sigma }\right\}\\ & \equiv & e^{-i\phi }\left\{I\cos\omega +i\sin\omega \hat{n}\cdot \hat{\sigma }\right\}, \end{array}$$

where the real parameter ω is determined from $\cos\omega \equiv \delta \delta^{\prime }+\tau \cdot \tau^{\prime }$. The unitarity condition of $\hat{u}$ can be checked by noting

(A32) $$\begin{array}{lll} \frac{1}{N^{2}} & = & {\left|\delta^{\prime }\tau -\delta \tau^{\prime }+\tau \times \tau^{\prime }\right|}^{2}\\ & = & {\left|\delta^{\prime }\tau -\delta \tau^{\prime }\right|}^{2}+\left(\tau \times \tau^{\prime }\right)\cdot \left(\tau \times \tau^{\prime }\right)\\ & = & \delta^{2}+\delta^{\prime 2}-2\delta \delta^{\prime }\tau \cdot \tau^{\prime }+\left(1-\delta^{2}\right)\left(1-\delta^{\prime 2}\right)-{\left(\tau \cdot \tau^{\prime }\right)}^{2}\\ & = & 1-{\left(\delta \delta^{\prime }+\tau \cdot \tau^{\prime }\right)}^{2}=1-\cos^{2}\omega , \end{array}$$

where we used the relation $\left(\tau \times \tau^{\prime }\right)\cdot \left(\tau \times \tau^{\prime }\right)={\left|\tau \right|}^{2}{\left|\tau^{\prime }\right|}^{2}-{\left(\tau \cdot \tau^{\prime }\right)}^{2}$. Now, the operator $\hat{u}+{\hat{u}}^{†}$ is calculated as

(A33) $$\begin{array}{ccc} \hat{u}+{\hat{u}}^{†} & = & 2\cos\phi \cos\omega I+2\sin\phi \sin\omega \hat{n}\cdot \hat{\sigma }, \end{array}$$

hence

(A34) $$\begin{array}{lll} \hat{W}{\hat{W}}^{†} & = & \left(\gamma_{b}^{2}+\gamma_{c}^{2}\right)I+2\gamma_{b}\gamma_{c}\left(\cos\phi \cos\omega I+\sin\phi \sin\omega \hat{n}\cdot \hat{\sigma }\right)\\ & \equiv & AI+B\cdot \hat{\sigma }. \end{array}$$

We defined

(A35) $$\begin{array}{ccc} A & = & \gamma_{b}^{2}+\gamma_{c}^{2}+2\gamma_{b}\gamma_{c}\cos\phi \cos\omega , \end{array}$$

(A36) $$\begin{array}{ccc} B & = & 2\gamma_{b}\gamma_{c}\sin\phi \sin\omega \hat{n}. \end{array}$$

Alternatively, we evaluate

(A37) $$\begin{array}{lll} {\hat{W}}^{†}\hat{W} & = & \left(\gamma_{b}{\hat{U}}_{b}^{†}+\gamma_{c}{\hat{U}}_{c}^{†}\right)\left(\gamma_{b}{\hat{U}}_{b}+\gamma_{c}{\hat{U}}_{c}\right)\\ & = & \left(\gamma_{b}^{2}+\gamma_{c}^{2}\right)I+\gamma_{b}\gamma_{c}\left({\hat{u}}^{\prime }+{\hat{u}}^{\prime †}\right), \end{array}$$

where ${\hat{u}}^{\prime }\equiv {\hat{U}}_{c}^{†}{\hat{U}}_{b}$, which represents the total development around the interferometer in the order 1→c→0→b→1. With similar procedure done for $\hat{u}$, we have

(A38) $$\begin{array}{lll} {\hat{u}}^{\prime } & = & e^{-i\phi }\left\{I\delta \delta^{\prime }+i\left(\delta^{\prime }\tau -\delta \tau^{\prime }\right)\cdot \hat{\sigma }+I\tau^{\prime }\cdot \tau +i\left(\tau^{\prime }\times \tau \right)\cdot \hat{\sigma }\right\}\\ & = & e^{-i\phi }\left\{I\cos\omega +i\sin\omega {\hat{n}}^{\prime }\cdot \hat{\sigma }\right\}, \end{array}$$

where

(A39) $$\begin{array}{ccc} {\hat{n}}^{\prime } & = & \frac{1}{\sqrt{1-\tau_{z}^{2}}}\left(\tau_{y},-\tau_{x},\delta \right), \end{array}$$

and with the same ω as before. Now, by calculating the factor ${\hat{u}}^{\prime }+{\hat{u}}^{\prime †}$, we obtain

(A40) $$\begin{array}{lll} {\hat{W}}^{†}\hat{W} & = & \left(\gamma_{b}^{2}+\gamma_{c}^{2}\right)I+2\gamma_{b}\gamma_{c}\left(\cos\phi \cos\omega I+\sin\phi \sin\omega {\hat{n}}^{\prime }\cdot \hat{\sigma }\right)\\ & \equiv & AI+B^{\prime }\cdot \hat{\sigma }, \end{array}$$

where

(A41) $$\begin{array}{ccc} B^{\prime } & = & 2\gamma_{b}\gamma_{c}\sin\phi \sin\omega {\hat{n}}^{\prime }. \end{array}$$

As shown in the main text, we introduce two sets of normalized eigenstates of the operators $\hat{n}\cdot \hat{\sigma }$ and ${\hat{n}}^{\prime }\cdot \hat{\sigma }$, $|\pm \hat{n}\rangle$ and $|\pm {\hat{n}}^{\prime }\rangle$, which obey

(A42) $$\begin{array}{ccc} \hat{W}{\hat{W}}^{†}|\pm \hat{n}\rangle & = & \lambda_{\pm }|\pm \hat{n}\rangle ,\\ {\hat{W}}^{†}\hat{W}|\pm {\hat{n}}^{\prime }\rangle & = & \lambda_{\pm }|\pm {\hat{n}}^{\prime }\rangle , \end{array}$$

with the same eigenvalues as Equation (19).

It can be shown that these two sets of eigenstates $\left\{|\pm \hat{n}\rangle ,|\pm {\hat{n}}^{\prime }\rangle \right\}$ are related with each other by

(A43) $$\begin{array}{ccc} |\pm {\hat{n}}^{\prime }\rangle & = & \frac{1}{\sqrt{\lambda_{\pm }}}{\hat{W}}^{†}|\pm \hat{n}\rangle , \end{array}$$

with double-sign correspondence. This can be checked by the eigen-equation

(A44) $$\begin{array}{lll} {\hat{W}}^{†}\hat{W}|\pm {\hat{n}}^{\prime }\rangle & = & \frac{1}{\sqrt{\lambda_{\pm }}}{\hat{W}}^{†}\left(\hat{W}{\hat{W}}^{†}\right)|\pm \hat{n}\rangle \\ & = & \frac{1}{\sqrt{\lambda_{\pm }}}{\hat{W}}^{†}\lambda_{\pm }|\pm \hat{n}\rangle \\ & = & \lambda_{\pm }|\pm {\hat{n}}^{\prime }\rangle , \end{array}$$

and the normalization condition

(A45) $$\begin{array}{lll} \langle \pm {\hat{n}}^{\prime }|\pm {\hat{n}}^{\prime }\rangle & = & \frac{1}{\lambda_{\pm }}\langle \pm \hat{n}\left|\hat{W}{\hat{W}}^{†}\right|\pm \hat{n}\rangle \\ & = & \langle \pm \hat{n}|\pm \hat{n}\rangle =1. \end{array}$$

Similarly, we can also prove the relation

(A46) $$\begin{array}{ccc} |\pm \hat{n}\rangle & = & \frac{1}{\sqrt{\lambda_{\pm }}}\hat{W}|\pm {\hat{n}}^{\prime }\rangle . \end{array}$$

We have the spectral decomposition of the matrices ${\hat{W}}^{†}$ and $\hat{W}$ by

(A47) $$\begin{array}{ccc} {\hat{W}}^{†} & = & \sqrt{\lambda_{+}}|{\hat{n}}^{\prime }\rangle \langle \hat{n}|+\sqrt{\lambda_{-}}|-{\hat{n}}^{\prime }\rangle \langle -\hat{n}|, \end{array}$$

(A48) $$\begin{array}{ccc} \hat{W} & = & \sqrt{\lambda_{+}}|\hat{n}\rangle \langle {\hat{n}}^{\prime }|+\sqrt{\lambda_{-}}|-\hat{n}\rangle \langle -{\hat{n}}^{\prime }|, \end{array}$$

where the second relation is obtained by taking Hermite conjugate of the first relation.

Now, let us turn to discuss the scattering wavefunctions using these eigenstates. For the left incoming states, we choose $|\chi_{\mathrm{in}}\rangle =|\pm \hat{n}\rangle$, then the transmitted states are

(A49) $$\begin{array}{lll} |\chi_{t,\pm }\rangle & = & \hat{t}|\pm \hat{n}\rangle \\ & = & i\eta_{k}{\hat{W}}^{†}{\left[IY-\hat{W}{\hat{W}}^{†}\right]}^{-1}|\pm \hat{n}\rangle \\ & = & i\eta_{k}{\hat{W}}^{†}{\left[IY-I\lambda_{\pm }\right]}^{-1}|\pm \hat{n}\rangle \\ & = & \frac{i\eta_{k}}{Y-\lambda_{\pm }}{\hat{W}}^{†}|\pm \hat{n}\rangle \\ & = & \frac{i\eta_{k}}{Y-\lambda_{\pm }}\sqrt{\lambda_{\pm }}|\pm {\hat{n}}^{\prime }\rangle \\ & = & t_{\pm }|\pm {\hat{n}}^{\prime }\rangle , \end{array}$$

where we used Equations (5) and (A47) and defined two transmission amplitudes,

(A50) $$\begin{array}{ccc} t_{\pm } & \equiv & \frac{i\eta_{k}\sqrt{\lambda_{\pm }}}{Y-\lambda_{\pm }}. \end{array}$$

Using the orthogonality of the eigenstates, the transmission amplitude matrix $\hat{t}$ is expressed as in Equation (24). Similarly, the reflected states are

(A51) $$\begin{array}{ccc} |\chi_{r,\pm }\rangle & = & \hat{r}|\pm \hat{n}\rangle =r_{\pm }|\pm \hat{n}\rangle , \end{array}$$

where the reflection amplitudes are

(A52) $$\begin{array}{ccc} r_{\pm } & \equiv & -1+\frac{i\eta_{k}X_{1}}{Y-\lambda_{\pm }}.\; \end{array}$$

The reflection amplitude matrix $\hat{r}$ is diagonal and is given in Equation (27). Similarly, for the right incoming states, the transmitted states are

(A53) $$\begin{array}{ccc} |\chi_{t,\pm }^{\prime }\rangle & = & {\hat{t}}^{\prime }|\pm {\hat{n}}^{\prime }\rangle =t_{\pm }|\pm \hat{n}\rangle , \end{array}$$

and hence the transmission amplitude matrix ${\hat{t}}^{\prime }$ is given in Equation (25). The reflected states are

(A54) $$\begin{array}{ccc} |\chi_{r,\pm }^{\prime }\rangle & = & {\hat{r}}^{\prime }|\pm {\hat{n}}^{\prime }\rangle =r_{\pm }^{\prime }|\pm {\hat{n}}^{\prime }\rangle , \end{array}$$

where we defined

(A55) $$\begin{array}{ccc} r_{\pm }^{\prime } & \equiv & -1+\frac{i\eta_{k}X_{0}}{Y-\lambda_{\pm }}, \end{array}$$

and hence the reflection amplitude matrix ${\hat{r}}^{\prime }$ is given in Equation (28).

## Appendix C. Unitarity of the Scattering Matrix

The scattering matrix needs to satisfy the unitarity condition, such that

(A56) $$\begin{array}{ccc} {\hat{t}}^{†}\hat{t}+{\hat{r}}^{†}\hat{r} & = & I. \end{array}$$

Using the results of Equations (24) and (27),

$$\begin{array}{ccc} {\hat{t}}^{†}\hat{t}+{\hat{r}}^{†}\hat{r} & = & {\left|t_{+}\right|}^{2}|\hat{n}\rangle \langle \hat{n}|+{\left|t_{-}\right|}^{2}|-\hat{n}\rangle \langle -\hat{n}|+{\left|r_{+}\right|}^{2}|\hat{n}\rangle \langle \hat{n}|+{\left|r_{-}\right|}^{2}|-\hat{n}\rangle \langle -\hat{n}|. \end{array}$$

Therefore, if ${\left|t_{\pm }\right|}^{2}+{\left|r_{\pm }\right|}^{2}=1$, using the completeness relation of $|\pm \hat{n}\rangle$, the unitarity is confirmed. Let us check this:

$$\begin{array}{ll} & {\left|t_{\pm }\right|}^{2}+{\left|r_{\pm }\right|}^{2}={\left|\frac{i\eta_{k}\sqrt{\lambda_{\pm }}}{Y-\lambda_{\pm }}\right|}^{2}+{\left|-1+\frac{i\eta_{k}X_{1}}{Y-\lambda_{\pm }}\right|}^{2}\\ = & \frac{\eta_{k}^{2}\lambda_{\pm }}{{\left|Y-\lambda_{\pm }\right|}^{2}}+1+\frac{i\eta_{k}X_{1}^{*}}{Y^{*}-\lambda_{\pm }}-\frac{i\eta_{k}X_{1}}{Y-\lambda_{\pm }}+\frac{\eta_{k}^{2}{\left|X_{1}\right|}^{2}}{{\left|Y-\lambda_{\pm }\right|}^{2}}\\ = & 1+i\eta_{k}\frac{X_{1}^{*}\left(Y-\lambda_{\pm }\right)-X_{1}\left(Y^{*}-\lambda_{\pm }\right)}{{\left|Y-\lambda_{\pm }\right|}^{2}}+\frac{\eta_{k}^{2}\left(\lambda_{\pm }+{\left|X_{1}\right|}^{2}\right)}{{\left|Y-\lambda_{\pm }\right|}^{2}}\\ = & 1+\frac{\eta_{k}}{{\left|Y-\lambda_{\pm }\right|}^{2}}\left[i\left\{X_{1}^{*}\left(Y-\lambda_{\pm }\right)-X_{1}\left(Y^{*}-\lambda_{\pm }\right)\right\}+\eta_{k}\left(\lambda_{\pm }+{\left|X_{1}\right|}^{2}\right)\right]. \end{array}$$

The factor in the square bracket is

$$\begin{array}{lll} \left[•\right] & = & -i\left(X_{1}^{*}-X_{1}\right)\lambda_{\pm }+i\left(X_{0}{\left|X_{1}\right|}^{2}-X_{0}^{*}{\left|X_{1}\right|}^{2}\right)+\eta_{k}\left(\lambda_{\pm }+{\left|X_{1}\right|}^{2}\right)\\ & = & \left[-i\left(-i\eta_{k}\right)+\eta_{k}\right]\lambda_{\pm }+\left[i\left(i\eta_{k}\right)+\eta_{k}\right]{\left|X_{1}\right|}^{2}=0, \end{array}$$

hence ${\left|t_{\pm }\right|}^{2}+{\left|r_{\pm }\right|}^{2}=1$ is confirmed.

## Appendix D. Derivatives of the Scattering Amplitude Matrices

In this Appendix, we evaluate the Berry curvature, Equation (33), with two control parameters, $g_{1},g_{2}$, which only modify the scattering eigenvalues $\lambda_{\pm }$ and corresponding eigenvectors $|\pm \hat{n}\rangle ,|\pm {\hat{n}}^{\prime }\rangle$. We need to calculate the derivatives of the scattering amplitude matrices by a control parameter ($g=g_{1},g_{2}$), $\frac{\partial \hat{r}}{\partial g}$ and $\frac{\partial {\hat{t}}^{\prime }}{\partial g}$. Since scattering amplitude matrices are expressed with the eigenstates as shown in Equations (24) and (27), we first evaluate the first order derivative of the basis states

(A57) $$\begin{array}{ccc} \frac{\partial }{\partial g}\left(\begin{array}{c} |\hat{n}\rangle \\ |-\hat{n}\rangle \end{array}\right) & = & \left(\begin{array}{cc} a_{g} & b_{g}\\ {\tilde{b}}_{g} & {\tilde{a}}_{g} \end{array}\right)\left(\begin{array}{c} |\hat{n}\rangle \\ |-\hat{n}\rangle \end{array}\right), \end{array}$$

(A58) $$\begin{array}{ccc} \frac{\partial }{\partial g}\left(\begin{array}{c} |{\hat{n}}^{\prime }\rangle \\ |-{\hat{n}}^{\prime }\rangle \end{array}\right) & = & \left(\begin{array}{cc} a_{g^{\prime }} & b_{g^{\prime }}\\ {\tilde{b}}_{g^{\prime }} & {\tilde{a}}_{g^{\prime }} \end{array}\right)\left(\begin{array}{c} |{\hat{n}}^{\prime }\rangle \\ |-{\hat{n}}^{\prime }\rangle \end{array}\right). \end{array}$$

Since the basis states are normalized,

(A59) $$\begin{array}{ccc} \frac{\partial }{\partial g}\langle \hat{n}|\hat{n}\rangle & = & \left(\frac{\partial \langle \hat{n}|}{\partial g}\right)|\hat{n}\rangle +\langle \hat{n}|\frac{\partial |\hat{n}\rangle }{\partial g}=a_{g}^{*}+a_{g}=0, \end{array}$$

and $a_{g}$ should be pure imaginary. Similarly, ${\tilde{a}}_{g}$, $a_{g^{\prime }}$ and ${\tilde{a}}_{g^{\prime }}$ are also pure imaginary. Using the orthogonality condition, we have the relation

(A60) $$\begin{array}{ccc} \frac{\partial }{\partial g}\langle \hat{n}|-\hat{n}\rangle & = & \left(\frac{\partial \langle \hat{n}|}{\partial g}\right)|-\hat{n}\rangle +\langle \hat{n}|\frac{\partial |-\hat{n}\rangle }{\partial g}=b_{g}^{*}+{\tilde{b}}_{g}=0, \end{array}$$

and $b_{g^{\prime }}^{*}+{\tilde{b}}_{g^{\prime }}=0$.

To have the formula for $a_{g}$, $b_{g}$ and ${\tilde{b}}_{g}$, we consider a unit vector $\hat{n}\equiv \left(n_{x},n_{y},n_{z}\right)=\left(\sin\Theta \cos\Phi ,\sin\Theta \sin\Phi ,\cos\Theta \right)$, with angles Θ and Φ. Since the operator $\hat{n}\cdot \hat{\sigma }$ in the matrix form,

(A61) $$\begin{array}{ccc} \hat{n}\cdot \hat{\sigma } & = & \left(\begin{array}{cc} \cos\Theta & \sin\Theta e^{-i\Phi }\\ \sin\Theta e^{i\Phi } & -\cos\Theta \end{array}\right), \end{array}$$

has two eigenvalues λ=±1, the eigenvector in a form ${\left(c_{1},c_{2}\right)}^{t}$ satisfies for λ=1, $\left(\cos\Theta -1\right)c_{1}+\left(\sin\Theta e^{-i\Phi }\right)c_{2}=0$ and normalization condition, and hence

(A62) $$\begin{array}{ccc} |n\rangle & = & {\left(\frac{n_{x}-in_{y}}{\sqrt{2(1-n_{z})}},\frac{\sqrt{1-n_{z}}}{\sqrt{2}}\right)}^{t}, \end{array}$$

and for λ=−1, $\left(\cos\Theta +1\right)c_{1}+\left(\sin\Theta e^{-i\Phi }\right)c_{2}=0$, hence

(A63) $$\begin{array}{ccc} |-n\rangle & = & {\left(\frac{-n_{x}+in_{y}}{\sqrt{2(1+n_{z})}},\frac{\sqrt{1+n_{z}}}{\sqrt{2}}\right)}^{t}. \end{array}$$

Differentiation with *g* gives

(A64) $$\begin{array}{ccc} \frac{\partial }{\partial g}|n\rangle & = & {\left(\frac{\frac{\partial n_{x}}{\partial g}-i\frac{\partial n_{y}}{\partial g}}{\sqrt{2(1-n_{z})}}+\frac{\left(n_{x}-in_{y}\right)\frac{\partial n_{z}}{\partial g}}{2\sqrt{2}{\left(1-n_{z}\right)}^{3/2}},-\frac{\frac{\partial n_{z}}{\partial g}}{2\sqrt{2}\sqrt{1-n_{z}}}\right)}^{t}, \end{array}$$

and

(A65) $$\begin{array}{ccc} \frac{\partial }{\partial g}|-n\rangle & = & {\left(\frac{-\frac{\partial n_{x}}{\partial g}+i\frac{\partial n_{y}}{\partial g}}{\sqrt{2(1-n_{z})}}+\frac{\left(n_{x}-in_{y}\right)\frac{\partial n_{z}}{\partial g}}{2\sqrt{2}{\left(1+n_{z}\right)}^{3/2}},\frac{\frac{\partial n_{z}}{\partial g}}{2\sqrt{2}\sqrt{1+n_{z}}}\right)}^{t}. \end{array}$$

Therefore,

(A66) $$\begin{array}{ccc} a_{g} & \equiv & \langle n|\frac{\partial }{\partial g}|n\rangle =\frac{i}{2(1-n_{z})}\left\{n_{y}\frac{\partial n_{x}}{\partial g}-n_{x}\frac{\partial n_{y}}{\partial g}\right\}, \end{array}$$

(A67) $$\begin{array}{ccc} {\tilde{a}}_{g} & \equiv & \langle -n|\frac{\partial }{\partial g}|-n\rangle =\frac{i}{2(1+n_{z})}\left\{n_{y}\frac{\partial n_{x}}{\partial g}-n_{x}\frac{\partial n_{y}}{\partial g}\right\},\\ b_{g} & \equiv & \langle -n|\frac{\partial }{\partial g}|n\rangle \end{array}$$

(A68) $$\begin{array}{lll} & = & -\frac{1}{2\sqrt{1-n_{z}^{2}}}\left\{in_{y}\frac{\partial n_{x}}{\partial g}-in_{x}\frac{\partial n_{y}}{\partial g}+\frac{\partial n_{z}}{\partial g}\right\},\\ {\tilde{b}}_{g} & \equiv & \langle n|\frac{\partial }{\partial g}|-n\rangle =-b_{g}^{*} \end{array}$$

(A69) $$\begin{array}{lll} & = & \frac{1}{2\sqrt{1-n_{z}^{2}}}\left\{-in_{y}\frac{\partial n_{x}}{\partial g}+in_{x}\frac{\partial n_{y}}{\partial g}+\frac{\partial n_{z}}{\partial g}\right\}. \end{array}$$

We also evaluate $a_{g^{\prime }},{\tilde{a}}_{g^{\prime }},b_{g^{\prime }}$ and ${\tilde{b}}_{g^{\prime }}$ similarly. From Equations (A30) and (A39), we found that following relations hold: $a_{g^{\prime }}=a_{g}$, ${\tilde{a}}_{g^{\prime }}={\tilde{a}}_{g}$ and $b_{g^{\prime }}=b_{g}^{*}$ and ${\tilde{b}}_{g^{\prime }}=-b_{g}$.

Using these relations, we evaluate the derivatives of the scattering amplitudes:

(A70) $$\begin{array}{lll} \frac{\partial \hat{r}}{\partial g} & = & \frac{\partial r_{+}}{\partial g}|\hat{n}\rangle \langle \hat{n}|+r_{+}\frac{\partial |\hat{n}\rangle }{\partial g}\langle \hat{n}|+r_{+}|\hat{n}\rangle \frac{\partial \langle \hat{n}|}{\partial g}\\ & & +\frac{\partial r_{-}}{\partial g}|-\hat{n}\rangle \langle -\hat{n}|+r_{-}\frac{\partial |-\hat{n}\rangle }{\partial g}\langle -\hat{n}|+r_{-}|-\hat{n}\rangle \frac{\partial \langle -\hat{n}|}{\partial g}\\ & \equiv & \left(|\hat{n}\rangle ,|-\hat{n}\rangle \right){\hat{r}}_{g}\left(\begin{array}{c} \langle \hat{n}|\\ \langle -\hat{n}| \end{array}\right), \end{array}$$

with defining a matrix

(A71) $$\begin{array}{ccc} {\hat{r}}_{g} & = & \left(\begin{array}{cc} \frac{\partial r_{+}}{\partial g} & \left(r_{+}-r_{-}\right)b_{g}^{*}\\ \left(r_{+}-r_{-}\right)b_{g} & \frac{\partial r_{-}}{\partial g} \end{array}\right). \end{array}$$

Similarly,

(A72) $$\begin{array}{lll} \frac{\partial {\hat{t}}^{\prime }}{\partial g} & = & \frac{\partial t_{+}}{\partial g}|\hat{n}\rangle \langle {\hat{n}}^{\prime }|+t_{+}\frac{\partial |\hat{n}\rangle }{\partial g}\langle {\hat{n}}^{\prime }|+t_{+}|\hat{n}\rangle \frac{\partial \langle {\hat{n}}^{\prime }|}{\partial g}\\ & + & \frac{\partial t_{-}}{\partial g}|-\hat{n}\rangle \langle -{\hat{n}}^{\prime }|+t_{-}\frac{\partial |-\hat{n}\rangle }{\partial g}\langle -{\hat{n}}^{\prime }|+t_{-}|-\hat{n}\rangle \frac{\partial \langle -{\hat{n}}^{\prime }|}{\partial g}\\ & \equiv & \left(|\hat{n}\rangle ,|-\hat{n}\rangle \right){\hat{t}}_{g}^{\prime }\left(\begin{array}{c} \langle {\hat{n}}^{\prime }|\\ \langle -{\hat{n}}^{\prime }| \end{array}\right), \end{array}$$

with defining a matrix

(A73) $$\begin{array}{ccc} {\hat{t}}_{g}^{\prime } & = & \left(\begin{array}{cc} \frac{\partial t_{+}}{\partial g} & t_{+}b_{g}-t_{-}b_{g}^{*}\\ t_{+}b_{g}-t_{-}b_{g}^{*} & \frac{\partial t_{-}}{\partial g} \end{array}\right). \end{array}$$

Obviously, it is convenient to take the scattering eigenstates $|\pm \hat{n}\rangle$ to the spin axis |σ=±1〉 in the Berry curvatures, which is written as

(A74) $$\begin{array}{ccc} \prod_{\hat{n}/-\hat{n}}\left(g_{1},g_{2}\right) & = & \frac{1}{\pi }ℑ\left\{{\left({\hat{r}}_{g_{2}}{\hat{r}}_{g_{1}}^{†}+{\hat{t}}_{g_{2}}^{\prime }{\hat{t}}_{g_{1}}^{\prime †}\right)}_{\left(1,1)/(2,2\right)}\right\}. \end{array}$$

Then the diagonal components of ${\hat{r}}_{g_{2}}{\hat{r}}_{g_{1}}^{†}$ is

(A75) $$\begin{array}{ccc} {\left({\hat{r}}_{g_{2}}{\hat{r}}_{g_{1}}^{†}\right)}_{\left(1,1\right)} & = & \frac{\partial r_{+}}{\partial g_{2}}\frac{\partial r_{+}^{*}}{\partial g_{1}}+{\left|r_{+}-r_{-}\right|}^{2}b_{g_{2}}^{*}b_{g_{1}}, \end{array}$$

(A76) $$\begin{array}{ccc} {\left({\hat{r}}_{g_{2}}{\hat{r}}_{g_{1}}^{†}\right)}_{\left(2,2\right)} & = & \frac{\partial r_{-}}{\partial g_{2}}\frac{\partial r_{-}^{*}}{\partial g_{1}}+{\left|r_{+}-r_{-}\right|}^{2}b_{g_{2}}b_{g_{1}}^{*}, \end{array}$$

and the diagonal components of ${\hat{t}}_{g_{2}}^{\prime }{\hat{t}}_{g_{1}}^{\prime †}$ is

(A77) $$\begin{array}{ccc} {\left({\hat{t}}_{g_{2}}^{\prime }{\hat{t}}_{g_{1}}^{\prime †}\right)}_{\left(1,1\right)} & = & \frac{\partial t_{+}}{\partial g_{2}}\frac{\partial t_{+}^{*}}{\partial g_{1}}+\left(t_{+}b_{g_{2}}-t_{-}b_{g_{2}}^{*}\right)\left(t_{+}^{*}b_{g_{1}}^{*}-t_{-}^{*}b_{g_{1}}\right), \end{array}$$

(A78) $$\begin{array}{ccc} {\left({\hat{t}}_{g_{2}}^{\prime }{\hat{t}}_{g_{1}}^{\prime †}\right)}_{\left(2,2\right)} & = & \frac{\partial t_{-}}{\partial g_{2}}\frac{\partial t_{-}^{*}}{\partial g_{1}}+\left(t_{+}b_{g_{2}}-t_{-}b_{g_{2}}^{*}\right)\left(t_{+}^{*}b_{g_{1}}^{*}-t_{-}^{*}b_{g_{1}}\right). \end{array}$$

As stated before, we restrict the type of control parameters, *g*, that only change the eigenvalues $\lambda_{\pm }$ in the transmission amplitudes, $r_{\pm }$ and $t_{\pm }$ and corresponding eigenstates, and we take

(A79) $$\begin{array}{cc} \frac{\partial r_{\pm }}{\partial g}=\frac{\partial \lambda_{\pm }}{\partial g}\frac{\partial r_{\pm }}{\partial \lambda_{\pm }},\;\; & \;\;\frac{\partial t_{\pm }}{\partial g}=\frac{\partial \lambda_{\pm }}{\partial g}\frac{\partial t_{\pm }}{\partial \lambda_{\pm }}. \end{array}$$

Then the factors in the Berry curvature, Equations (A75) and (A77),

(A80) $$\begin{array}{ccc} \frac{\partial r_{\pm }}{\partial g_{2}}\frac{\partial r_{\pm }^{*}}{\partial g_{1}} & = & \frac{\partial \lambda_{\pm }}{\partial g_{2}}\frac{\partial \lambda_{\pm }}{\partial g_{1}}{\left|\frac{\partial r_{\pm }}{\partial \lambda_{\pm }}\right|}^{2}, \end{array}$$

(A81) $$\begin{array}{ccc} \frac{\partial t_{\pm }}{\partial g_{2}}\frac{\partial t_{\pm }^{*}}{\partial g_{1}} & = & \frac{\partial \lambda_{\pm }}{\partial g_{2}}\frac{\partial \lambda_{\pm }}{\partial g_{1}}{\left|\frac{\partial t_{\pm }}{\partial \lambda_{\pm }}\right|}^{2}, \end{array}$$

are real and are not contributing to the pumping.

Then, the Berry curvature for the spin $|\hat{n}\rangle$ is

(A82) $$\begin{array}{lll} \prod_{\hat{n}}\left(g_{1},g_{2}\right) & = & \frac{1}{\pi }ℑ\left\{{\left|r_{+}-r_{-}\right|}^{2}b_{g_{2}}^{*}b_{g_{1}}+\left(t_{+}b_{g_{2}}-t_{-}b_{g_{2}}^{*}\right)\left(t_{+}^{*}b_{g_{1}}^{*}-t_{-}^{*}b_{g_{1}}\right)\right\}\\ & = & \frac{1}{2\pi i}\left({\left|r_{+}-r_{-}\right|}^{2}-{\left|t_{+}\right|}^{2}+{\left|t_{-}\right|}^{2}\right)\left(b_{g_{2}}^{*}b_{g_{1}}-b_{g_{2}}b_{g_{1}}^{*}\right). \end{array}$$

Using Equation (A68) and the relation

(A83) $$\begin{array}{ccc} \frac{\partial n_{z}}{\partial g} & = & -\frac{1}{n_{z}}\left(n_{x}\frac{\partial n_{x}}{\partial g}+n_{y}\frac{\partial n_{y}}{\partial g}\right), \end{array}$$

derived from $n_{z}=\sqrt{1-n_{x}^{2}-n_{y}^{2}}$, the factor in the last bracket of Equation (A82) is manipulated to

(A84) $$\begin{array}{ccc} b_{g_{2}}^{*}b_{g_{1}}-b_{g_{2}}b_{g_{1}}^{*} & = & \frac{i}{2n_{z}}\left(\frac{\partial n_{x}}{\partial g_{2}}\frac{\partial n_{y}}{\partial g_{1}}-\frac{\partial n_{x}}{\partial g_{1}}\frac{\partial n_{y}}{\partial g_{2}}\right)\equiv 2\pi iC_{g_{1},g_{2}}. \end{array}$$

From Equation (A30), the derivatives of the elements of $\hat{n}$ by some control parameter *g* are calculated as

(A85) $$\begin{array}{ccc} \frac{\partial n_{x}}{\partial g} & = & -\frac{1}{\sqrt{1-\tau_{z}^{2}}}\left\{\frac{\partial \tau_{y}}{\partial g}+\frac{\tau_{y}\tau_{z}}{1-\tau_{z}^{2}}\frac{\partial \tau_{z}}{\partial g}\right\}, \end{array}$$

(A86) $$\begin{array}{ccc} \frac{\partial n_{y}}{\partial g} & = & \frac{1}{\sqrt{1-\tau_{z}^{2}}}\left\{\frac{\partial \tau_{x}}{\partial g}+\frac{\tau_{x}\tau_{z}}{1-\tau_{z}^{2}}\frac{\partial \tau_{z}}{\partial g}\right\}, \end{array}$$

therefore, the factor $C_{g_{1},g_{2}}$ in Equation (A84) is obtained as Equation (36) and the Berry curvatures are given as Equations (34) and (35).

## Appendix E. Formulation of Diamond-Shape Interferometer

This section explains the foundation of the Schrödinger Equations (3) and (4). At the four sites in the interferometer, the Schrödinger equation is

(A87) $$\begin{array}{ccc} (\epsilon -\epsilon_{u})|\psi (u)\rangle & = & -\sum_{v}{\tilde{U}}_{uv}|\psi (v)\rangle . \end{array}$$

Explicitly, at sites u=0,1,b,c:

(A88) $$\begin{array}{ccc} (\epsilon -\epsilon_{0})|\psi (0)\rangle & = & -\left({\tilde{U}}_{0b}|\psi (b)\rangle +{\tilde{U}}_{0c}|\psi (c)\rangle \right)-j|\psi (-1)\rangle , \end{array}$$

(A89) $$\begin{array}{ccc} (\epsilon -\epsilon_{1})|\psi (1)\rangle & = & -\left({\tilde{U}}_{b1}^{†}|\psi (b)\rangle +{\tilde{U}}_{c1}^{†}|\psi (c)\rangle \right)-j|\psi (2)\rangle , \end{array}$$

(A90) $$\begin{array}{ccc} (\epsilon -\epsilon_{b})|\psi (b)\rangle & = & -\left({\tilde{U}}_{0b}^{†}|\psi (0)\rangle +{\tilde{U}}_{b1}|\psi (1)\rangle \right), \end{array}$$

(A91) $$\begin{array}{ccc} (\epsilon -\epsilon_{c})|\psi (c)\rangle & = & -\left({\tilde{U}}_{0c}^{†}|\psi (0)\rangle +{\tilde{U}}_{c1}|\psi (1)\rangle \right). \end{array}$$

Using Equations (A90) and (A91),

(A92) $$\begin{array}{ccc} |\psi (b)\rangle & = & -\frac{1}{\epsilon -\epsilon_{b}}\left[{\tilde{U}}_{0b}^{†}|\psi (0)\rangle +{\tilde{U}}_{b1}|\psi (1)\rangle \right], \end{array}$$

(A93) $$\begin{array}{ccc} |\psi (c)\rangle & = & -\frac{1}{\epsilon -\epsilon_{c}}\left[{\tilde{U}}_{0c}^{†}|\psi (0)\rangle +{\tilde{U}}_{c1}|\psi (1)\rangle \right]. \end{array}$$

By putting these into Equation (A88),

$$\begin{array}{lll} (\epsilon -\epsilon_{0})|\psi (0)\rangle & = & -{\tilde{U}}_{0b}\left(-\frac{1}{\epsilon -\epsilon_{b}}\right)\left[{\tilde{U}}_{0b}^{†}|\psi (0)\rangle +{\tilde{U}}_{b1}|\psi (1)\rangle \right]\\ & & -{\tilde{U}}_{0c}\left(-\frac{1}{\epsilon -\epsilon_{c}}\right)\left[{\tilde{U}}_{0c}^{†}|\psi (0)\rangle +{\tilde{U}}_{c1}|\psi (1)\rangle \right]-j|\psi (-1)\rangle \\ & = & \left(\frac{J_{0b}J_{b0}}{\epsilon -\epsilon_{b}}+\frac{J_{0c}J_{c0}}{\epsilon -\epsilon_{c}}\right)|\psi (0)\rangle +\left[\frac{{\tilde{U}}_{0b}{\tilde{U}}_{b1}}{\epsilon -\epsilon_{b}}+\frac{{\tilde{U}}_{0c}{\tilde{U}}_{c1}}{\epsilon -\epsilon_{c}}\right]|\psi (1)\rangle -j|\psi (-1)\rangle . \end{array}$$

Then we define real variables

(A94) $$\begin{array}{ccc} \gamma_{uvw} & \equiv & \frac{J_{uv}J_{uw}}{\epsilon -\epsilon_{v}}, \end{array}$$

and introducing a 2×2 matrix

(A95) $$\begin{array}{lll} \hat{W} & \equiv & \frac{{\tilde{U}}_{0b}{\tilde{U}}_{b1}}{\epsilon -\epsilon_{b}}+\frac{{\tilde{U}}_{0c}{\tilde{U}}_{c1}}{\epsilon -\epsilon_{c}}\\ & = & \gamma_{0b1}{\hat{U}}_{0b}{\hat{U}}_{b1}+\gamma_{0c1}{\hat{U}}_{0c}{\hat{U}}_{c1}, \end{array}$$

we obtain the relation equivalent to Equation (3)

(A96) $$\begin{array}{ccc} \left(\epsilon -y_{0}\right)|\psi (0)\rangle & = & \hat{W}|\psi (1)\rangle -j|\psi (-1)\rangle , \end{array}$$

where we defined renormalized site energy at u=0, $y_{0}\equiv \epsilon_{0}+\gamma_{0b0}+\gamma_{0c0}$. By putting Equation (A92) in Equation (89),

$$\begin{array}{lll} (\epsilon -\epsilon_{1})|\psi (1)\rangle & = & {\tilde{U}}_{b1}^{†}\left(\frac{1}{\epsilon -\epsilon_{b}}\right)\left[{\tilde{U}}_{0b}^{†}|\psi (0)\rangle +{\tilde{U}}_{b1}|\psi (1)\rangle \right]\\ & & +{\tilde{U}}_{c1}^{†}\left(\frac{1}{\epsilon -\epsilon_{c}}\right)\left[{\tilde{U}}_{0c}^{†}|\psi (0)\rangle +{\tilde{U}}_{c1}|\psi (1)\rangle \right]-j|\psi (2)\rangle \\ & = & \left(\frac{J_{1b}J_{b0}}{\epsilon -\epsilon_{b}}{\hat{U}}_{b1}^{†}{\hat{U}}_{0b}^{†}+\frac{J_{1c}J_{c0}}{\epsilon -\epsilon_{c}}{\hat{U}}_{c1}^{†}{\hat{U}}_{0c}^{†}\right)|\psi (0)\rangle +\left(\frac{J_{1b}J_{b1}}{\epsilon -\epsilon_{b}}+\frac{J_{1c}J_{c1}}{\epsilon -\epsilon_{c}}\right)|\psi (1)\rangle -j|\psi (2)\rangle . \end{array}$$

Hence, we have the equation equivalent to Equation (4)

(A97) $$\begin{array}{ccc} (\epsilon -y_{1})|\psi (1)\rangle & = & {\hat{W}}^{†}|\psi (0)\rangle -j|\psi (2)\rangle , \end{array}$$

where we introduced renormalized site energy at u=1, $y_{1}\equiv \epsilon_{1}+\gamma_{1b1}+\gamma_{1c1}$. We defined $\gamma_{b}\equiv \gamma_{0b1}$, $\gamma_{c}\equiv \gamma_{0c1}$ and ${\hat{U}}_{b}\equiv {\hat{U}}_{0b}{\hat{U}}_{b1}$, ${\hat{U}}_{c}\equiv {\hat{U}}_{0c}{\hat{U}}_{c1}$.
